# Advances in LDI-MS Analysis: The Role of Chemical Vapor Deposition-Synthesized Silver Nanoparticles in Enhancing Detection of Low-Molecular-Weight Biomolecules

**DOI:** 10.1021/jasms.4c00071

**Published:** 2024-08-14

**Authors:** Ewelina Sibińska, Justyna Walczak-Skierska, Adrian Arendowski, Agnieszka Ludwiczak, Aleksandra Radtke, Piotr Piszczek, Dorota Gabryś, Kinga Robotnik, Paweł Pomastowski

**Affiliations:** † Centre for Modern Interdisciplinary Technologies, Nicolaus Copernicus University in Toruń, Wileńska 4 Str., 87-100 Toruń, Poland; ‡ Faculty of Biological and Veterinary Sciences, Nicolaus Copernicus University in Toruń, Lwowska 1 Str., 87-100 Toruń, Poland; § Department of Inorganic and Coordination Chemistry, Faculty of Chemistry, Nicolaus Copernicus University in Toruń, Gagarina 7 Str., 87-100 Toruń, Poland; ∥ Radiotherapy Department, Maria Sklodowska-Curie National Research Institute of Oncology, Wybrzeże Armii Krajowej 15 Str., 44-102 Gliwice, Poland

**Keywords:** laser desorption/ionization mass spectrometry, nanoparticle-based laser desorption/ionization, chemical vapor deposition, silver nanoparticles, triglycerides, saccharides

## Abstract

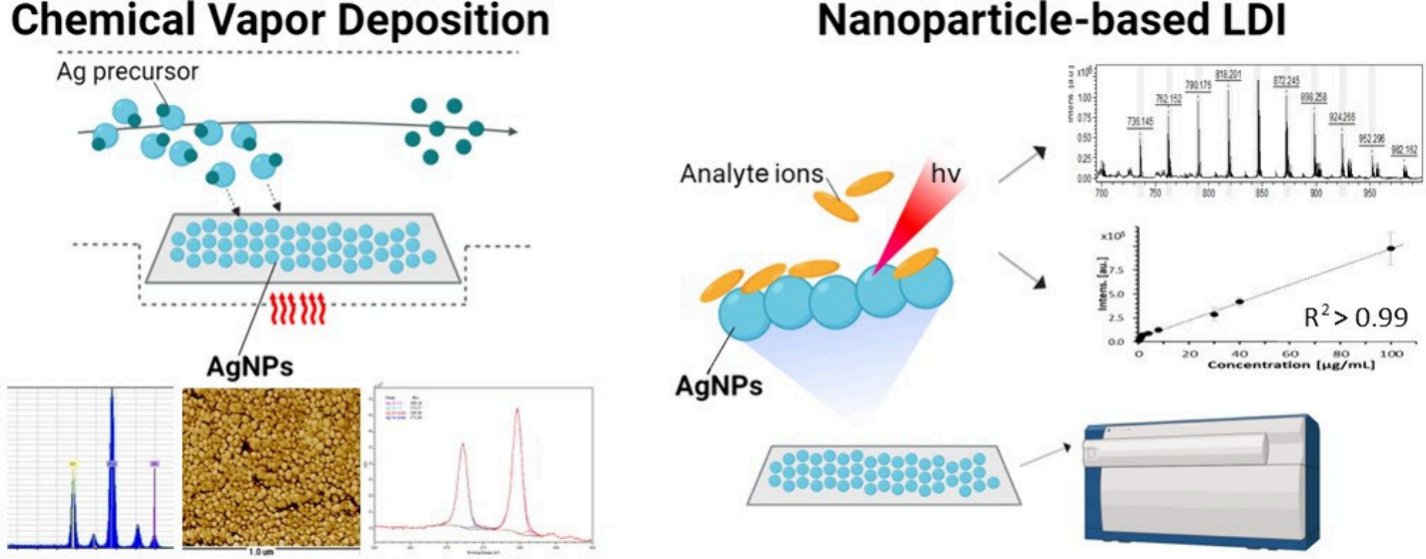

In this investigation, we detail the synthesis of silver nanoparticles (AgNPs) via a precise chemical vacuum deposition (CVD) methodology, aimed at augmenting the analytical performance of laser desorption/ionization mass spectrometry (LDI-MS) for the detection of low-molecular-weight analytes. Employing a precursor supply rate of 0.0014 mg/s facilitated the formation of uniformly dispersed AgNPs, characterized by SEM and AFM to have an average diameter of 33.5 ± 1.5 nm and a surface roughness (*R*_a_) of 11.8 nm, indicative of their homogeneous coverage and spherical morphology. XPS and SEM-EDX analyses confirmed the metallic silver composition of the nanoparticles with Ag peak splitting, reflecting the successful synthesis of metallic Ag. Comparative analytical evaluation with traditional MALDI matrices revealed that AgNPs significantly reduce signal suppression, thereby enhancing the sensitivity and specificity of LDI-MS for low-molecular-weight compounds such as triglycerides, saccharides, amino acids, and carboxylic acids. Notably, the application of AgNPs demonstrated a superior linear response for triglyceride signals with regression coefficients surpassing 0.99, markedly outperforming conventional matrices. The study further extends into quantitative analysis through nanoparticle-based laser desorption/ionization (NALDI), where AgNPs exhibited enhanced ionization efficiency, characterized by substantially lower limits of detection (LOD) and quantification (LOQ) for tested standards. Particular attention was paid to lipids with a detailed examination of their fragmentation pathways. These results highlight the significant potential of AgNPs synthesized via CVD to transform the analytical detection and quantification of low-molecular-weight compounds using NALDI. This approach offers a promising avenue for expanding the scope of analytical applications in mass spectrometry and introducing innovative methodologies for enhanced precision and sensitivity.

## 1 Introduction

Nanoparticles (NPs) are defined as structures with at least one dimension ranging from 1 to 100 nm.^1^ As the size of these particles decreases, the proportion of atoms on the surface relative to those inside the particle increases, leading to significant alterations in their physical and chemical properties. In comparison to their larger counterparts, NPs exhibit variations in a range of characteristics, including density, solubility, spectroscopic properties, melting points, surface tension, mechanical strengths, electrical and thermal conductivities, magnetic responses, crystalline structures, and catalytic activities.^2^ Noble metal nanoparticles, particularly those of silver (Ag) and gold (Au), are of paramount importance in various spectroscopic methods, owing to their distinct optical properties. These nanoparticles are capable of interacting with different forms of radiation, such as ultraviolet (UV), visible, and infrared (IR) light through mechanisms that include absorption, scattering, and surface-enhanced processes.

The valence electrons of atoms located on the surface of metal nanoparticles, known as surface plasmons, can absorb electromagnetic radiation. This absorption leads to the collective oscillation of these electrons, creating a coherent motion known as localized surface plasmon resonance (LSPR). The movement is influenced by the restoring forces associated with the positively charged nuclei and the overall electron cloud. After the cessation of the external stimulus, the oscillating electrons return to a state of equilibrium as a result of the attractive Coulombic forces between the electrons and the nuclei. To observe this phenomenon, the incident electromagnetic wave on the plasmonic nanoparticle must possess a frequency that matches the vibration frequency of the localized surface plasmons.^3−5^ This process is manifested by an increase in light extinction (absorption and scattering) as well as the creation of strong electromagnetic fields around the nanoparticle, which are responsible for the effectiveness of photocatalytic properties. Absorption of radiation with energy equal to or greater than the band gap energy causes the induction of electronic transitions in plasmonic metals (interband and intraband transitions). These electrons constitute charge carriers termed hot electrons, which can migrate to all available unoccupied states, including molecules adsorbed on the nanoparticle surface, through an indirect charge transfer pathway. This contributes to the direct photocatalysis of analytes adsorbed on the surface.^6^ The frequency can be adjusted by selecting the size, shape, and material type of the nanoparticles. In the case of small-sized silver and gold nanoparticles, luminescence tends to shift toward the blue spectrum, while larger ones exhibit a shift toward the red spectrum. These parameters allow for control over resonance and adaptation to desired wavelengths for planned applications.^7^

The unique optical properties of metallic NPs find application in various analytical techniques for detecting biological molecules. In surface spectroscopy, NPs serve to amplify spectroscopic signals, enhancing sensitivity and enabling the detection of substances, even at low concentrations. This is exemplified in techniques such as surface-enhanced Raman scattering (SERS),^8^ metal-enhanced fluorescence (MEF),^9^ and surface-enhanced infrared absorption (SEIRA).^10^ In mass spectrometry, metallic NPs act as matrices in nanoparticle-based laser desorption/ionization (NALDI), facilitating rapid and sensitive analysis of chemical compounds.^11^ This technique presents a promising alternative to matrix-assisted laser desorption/ionization (MALDI). Organic matrices employed in MALDI exhibit strong absorption of laser radiation, resulting in spectra cluttered with numerous signals originating from the matrix itself, its fragments, and various adducts. This abundance of signals complicates the interpretation of spectra, particularly for molecules with a mass below 500 Da.^12^ There is a compelling argument for the application of noble metal nanoparticles in LDI techniques, given their (i) relatively high tolerance to salts, (ii) elimination of suppression from matrix-related ions, (iii) generation of highly reproducible signals, and (iv) potential for internal calibration.^11^ Several papers have demonstrated the application of silver nanoparticles (AgNPs) in detecting various low-mass compounds in laser desorption/ionization mass spectrometry (LDI-MS), providing evidence of their value in the detection and quantification of pure compounds such as nucleosides and nucleic bases,^13^ carboxylic acids,^14^ lipids,^15^ and drug metabolites^16^ and the analysis of complex biological tissues with the use of MS imaging.^17^

The burgeoning interest in silver nanoparticles has placed the spotlight on their synthesis, stabilization, and characterization, marking these areas as hotbeds of intensive research in recent years. A pivotal aim in the advancement of nanotechnology is the continuous improvement and standardization of methods for nanomaterial synthesis and surface modification. Such efforts are crucial for generating materials that not only are more stable but also exhibit improved uniformity in shape and particle size. Metal nanoparticles are produced via various methods, broadly categorized into two main strategies: the top-down (destructive) method and the bottom-up (constructive) method. The bottom-up approach is particularly noteworthy for its enhanced control over the formation and chemical composition of the final product, a vital aspect for crafting materials with desired specific physicochemical properties.^18^ This category also encompasses chemical vapor deposition (CVD), a technique that, according to our prior studies, shows promise in the synthesis of silver nanoparticles for the analysis of low-molecular-weight compounds in mass spectrometry.^12,15^ In the CVD process, nanoparticles are formed on a substrate by sublimation of a precursor. This precursor is transported in gaseous form to the substrate, where it undergoes chemical reactions, typically thermal decomposition for metals, leading to the deposition of a thin nanoparticle layer.^19^

The aim of this study was to utilize the chemical vapor deposition (CVD) technique for synthesizing a uniform layer of silver nanoparticles (AgNPs) on a steel substrate and to conduct a comprehensive characterization of the resultant system. Following this, we assessed the feasibility of employing this system in laser desorption/ionization mass spectrometry (LDI-MS) for the analysis of low-molecular-weight compounds, such as lipids, saccharides, amino acids, and carboxylic acids. Significantly, this research presents for the first time the application potential of AgNPs synthesized via this method in the quantitative analysis of triglycerides (TGs), marking a novel contribution to the fields of analytical chemistry and nanotechnology.

## 2 Methodology

### 2.1 Synthesis of Silver Nanoparticles

Silver nanoparticle layers were synthesized by using the chemical vapor deposition (CVD) method, and a hot-wall reactor was employed in all deposition processes. The precursor for AgNPs was a chemical compound with the molecular formula Ag_5_(O_2_CC_2_F_5_)_5_(H_2_O)_3_ (solid state). In all processes, 5 mg of the precursor was applied, and the sublimation temperature (*T*_V_) and deposition temperature (*T*_D_) were set at 240 and 290 °C, respectively. Additionally, argon was used as the carrier gas, and the deposition time (*t*) was 60 min.

AgNP layers were fabricated on steel substrates (stainless steel H17). The substrate preparation process involved degreasing its surface using Viruton Extra, enabling washing and disinfection of steel substrates in an ultrasonic bath (3 × 15 min). Substrates were then rinsed in distilled water and stored in anhydrous ethanol (EtOH). Before the deposition process, the substrate was dried in a stream of Ar, and the surface was activated by immersion in a 0.1% solution of trifluoroacetic acid (TFA). After the substrate surface was dried in a stream of Ar, the sample was placed in the CVD reactor.

### 2.2 Characterization of the Obtained AgNPs

In order to perform analyses enabling the characterization of the obtained plates, nanoparticles were deposited under the same conditions on a substrate (H17 steel) with dimensions of 1 × 1 cm. The produced AgNP layers were characterized by using scanning electron microscopy (SEM), SEM energy dispersive X-ray spectroscopy (SEM-EDX), atomic force microscopy (AFM), X-ray photoelectron spectroscopy (XPS), ultraviolet–visible diffuse reflectance spectroscopy (UV–vis DRS), and Raman spectroscopy.

### 2.3 Sample Preparation

Analysis involved the use of standards of lipids (TG Internal Standard Mixture UltimateSPLASH ONE, 16:0 PG (phosphatidylglycerol), 18:0 PC (phosphatidylcholine); Avanti Polar Lipids), fatty acids (myristic acid and stearic acid; Sigma-Aldrich), saccharides (lactose and glucose; Sigma-Aldrich), amino acids (methionine and lysine; Sigma-Aldrich), thymidine (Sigma-Aldrich), and carboxylic acids (ascorbic acid and shikimic acid; Sigma-Aldrich). TG Internal Standard Mixture contained a mixture of nine triglycerides dissolved in a mixture of dichloromethane:methanol (vol, 1:1). The remaining standards were in the form of powder, which were weighed and dissolved in appropriate solvents (see Table 1) to obtain an initial concentration of 1 mg/mL. The solutions were applied to a steel plate coated with nanoparticles in a volume of 0.5 μL. After being dried, the target was inserted into the MS apparatus for measurements.

**Table 1:** List of Compound Standards Used for Research^a^

	compound formula	molar mass	concentration	solvent
TG internal standard	14:0-13:0-14:0 TG-d5 C_44_H_79_D_5_O_6_	713.66	25 μg/mL	dichloromethane:methanol (vol:vol, 1:1)
TG internal standard	14:0-15:1-14:0 TG-d5 C_46_H_81_D_5_O_6_	739.67	50 μg/mL	dichloromethane:methanol (vol:vol, 1:1)
TG internal standard	14:0-17:1-14:0 TG-d5 C_48_H_85_D_5_O_6_	767.71	75 μg/mL	dichloromethane:methanol (vol:vol, 1:1)
TG internal standard	16:0-15:1-16:0 TG-d5 C_50_H_89_D_5_O_6_	795.74	100 μg/mL	dichloromethane:methanol (vol:vol, 1:1)
TG internal standard	16:0-17:1-16:0 TG-d5 C_52_H_93_D_5_O_6_	823.77	125 μg/mL	dichloromethane:methanol (vol:vol, 1:1)
TG internal standard	16:0-19:2-16:0 TG-d5 C_54_H_95_D_5_O_6_	849.78	100 μg/mL	dichloromethane:methanol (vol:vol, 1:1)
TG internal standard	18:1-17:1-18:1 TG-d5 C_56_H_97_D_5_O_6_	875.80	75 μg/mL	dichloromethane:methanol (vol:vol, 1:1)
TG internal standard	18:1-19:2-18:1 TG-d5 C_58_H_99_D_5_O_6_	901.81	50 μg/mL	dichloromethane:methanol (vol:vol, 1:1)
TG internal standard	18:1-21:2-18:1 TG-d5 C_60_H_103_D_5_O_6_	929.85	25 μg/mL	dichloromethane:methanol (vol:vol, 1:1)
	16:0 PG C_38_H_74_O_10_PNa	744.95	1 mg/mL	dichloromethane:methanol (vol:vol, 1:1)
	18:0 PC C_44_H_88_NO_8_P	790.20	1 mg/mL	dichloromethane:methanol (vol:vol, 1:1)
	myristic acid C_14_H_28_O_2_	228.38	1 mg/mL	dichloromethane:methanol (vol:vol, 1:1)
	stearic acid C_18_H_36_O_2_	284.48	1 mg/mL	dichloromethane:methanol (vol:vol, 1:1)
	glucose C_6_H_12_O_6_	180.16	1 mg/mL	water
	lactose C_12_H_22_O_11_	342.30	1 mg/mL	water
	methionine C_5_H_11_NO_2_S	149.21	1 mg/mL	water
	lysine C_6_H_14_N_2_O_2_	146.19	1 mg/mL	water
	thymidine C_10_H_14_N_2_O_5_	242.23	1 mg/mL	water
	ascorbic acid C_6_H_8_O_6_	176.12	1 mg/mL	water
	shikimic acid C_7_H_10_O_5_	164.15	1 mg/mL	water

a Concentration refers to the initial concentration from which dilutions were subsequently made.

For selected standards, a series of dilutions were made, ensuring that the final standard solution contained 0.02 μg/mL. All prepared dilutions were plated on a steel plate coated with nanoparticles and a MALDI plate in a volume of 0.5 μL. In the case of the MALDI technique, a ground steel plate was used, and the samples were covered with two different matrices: α-cyano-4-hydroxycinnamic acid (HCCA, Bruker Daltonics, Bremen, Germany) and 2,5-dihydroxybenzoic acid (DHB, Bruker Daltonics, Bremen, Germany). The matrices were prepared by dissolving in a mixture of 50% acetonitrile, 47.5% water, and 2.5% trifluoroacetic acid (Bruker standard solvent, Merck, Warsaw, Poland). The HCCA matrix concentration was 10 mg/mL, and that of DHB was 20 mg/mL.

### 2.4 Sample Analysis via LDI-MS

LDI mass spectrometry experiments were performed in positive reflectron mode (ion source 1: 25.05 kV; ion source 2: 22.40 kV) using a Bruker ultrafleXtreme instrument equipped with a SmartBeam II laser (355 nm and frequency of 2 kHz). Each spot underwent a total of 10 000 laser shots. This number of laser shots was distributed among five points symmetrically positioned around the center of the spot. At each point, 2000 laser shots were conducted, employing the default random walk approach (random points with 50 laser shots each). The measurement range encompassed a mass-to-charge ratio (*m*/*z*) range of 100–2000, with suppression typically activated for ions with *m*/*z* values lower than 80. The data were calibrated and examined utilizing FlexAnalysis (version 3.3), employing a centroid peak detection algorithm. Mass calibration for a plate coated with silver nanoparticles relied on internal standards, utilizing signals from ^107^Ag and ^109^Ag ions, along with their Ag_2_ and Ag_3_ adducts (quadratic mode). For the MALDI technique, cesium iodide mixed with a DHB matrix was employed. The instrument parameters applied for both NALDI and MALDI were as follows: 60% laser power, detector gain set to 30×, a global attenuator value of 45%, the laser focus parameter set to “large”, a sensitivity of digitizer of 100 mV, an analog offset reflectron of 2.6 mV, and a trigger level of 800 mV. For quantitative analyses, four spectra were collected for each sample dilution. The silver nanostructured substrates were analyzed using an MTP Slide Adapter II adapter (Bruker Daltonics, Bremen, Germany). For quantitative analyses, four spectra were collected for each sample dilution (one data point). The limit of detection (LOD) was calculated based on a signal-to-noise (S/N) ratio of 3 according to ref (20), while the limit of quantification (LOQ) was calculated based on an S/N ratio of 5 according to ref (21). Therefore, signals for which S/N > 5 were included in the curve. At least five data points were required to plot a curve.

## 3 Results

### 3.1 Characterization of AgNPs

**Figure 1:**
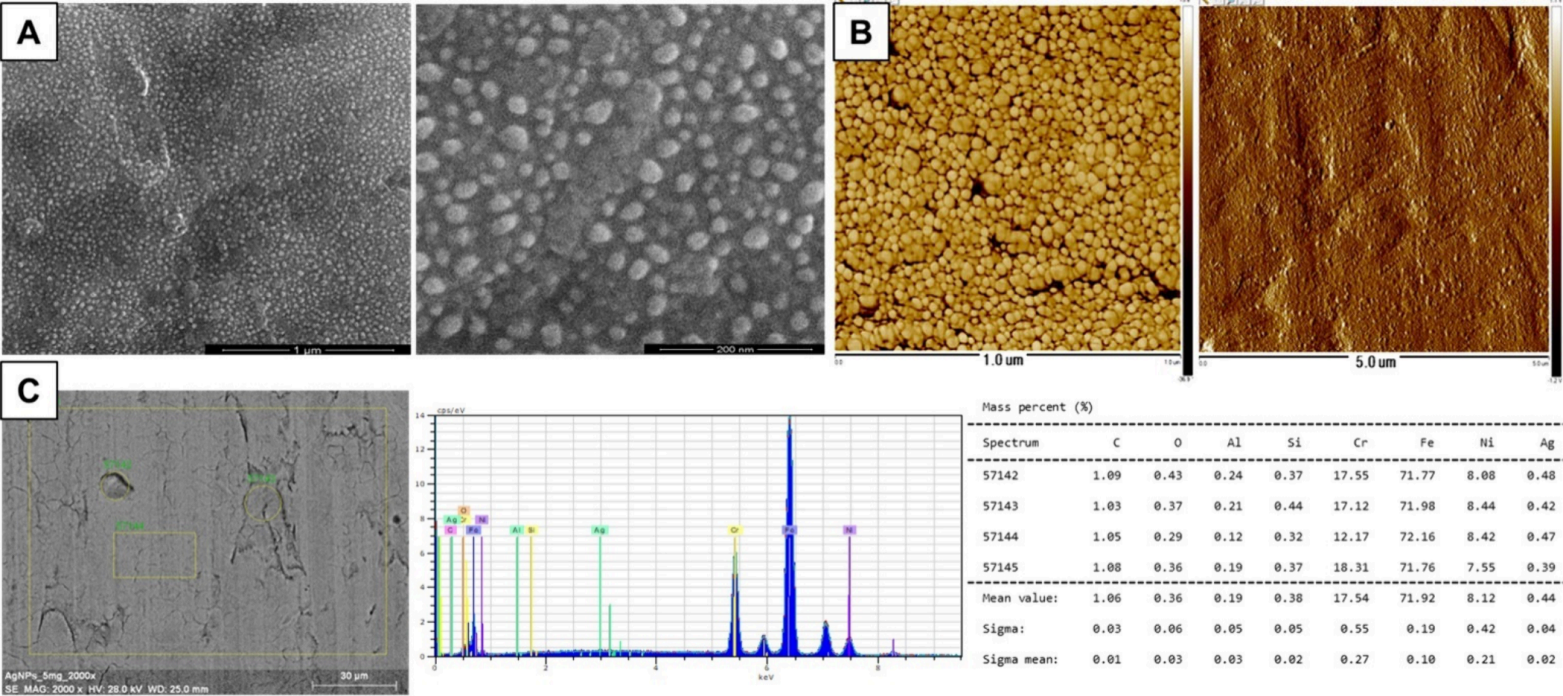
(A) Scanning electron microscopy and (B) atomic force microscopy (AFM) images of the AgNP layer deposited on the surface of stainless steel (H17) using the CVD technique. (C) SEM-EDX analysis of the AgNP layer.

Employing a precursor supply rate of 0.0014 mg/s in the chemical vapor deposition (CVD) reactor (totaling 5 mg over 60 min) led to the development of dispersed silver nanoparticle (AgNP) nuclei. Scanning electron microscopy (SEM) studies revealed a monolayer of AgNPs consisting of particles with an average diameter of 33.5 ± 1.5 nm, uniformly distributed across the entire surface (Figure 1A). Atomic force microscopy (AFM) images further corroborated the monolayer’s morphology, showcasing individual nanoparticles (Figure 1B). Both techniques confirmed the spherical shape of the nanoparticles. Analysis of the roughness parameters over a surface area of 1 μm^2^ yielded an average roughness (*R*_a_) of 11.8 nm and a root-mean-square roughness (*R*_q_) of 14.0 nm. SEM-EDX analysis verified the presence of AgNPs on the substrate surface (Figure 1C).

**Figure 2:**
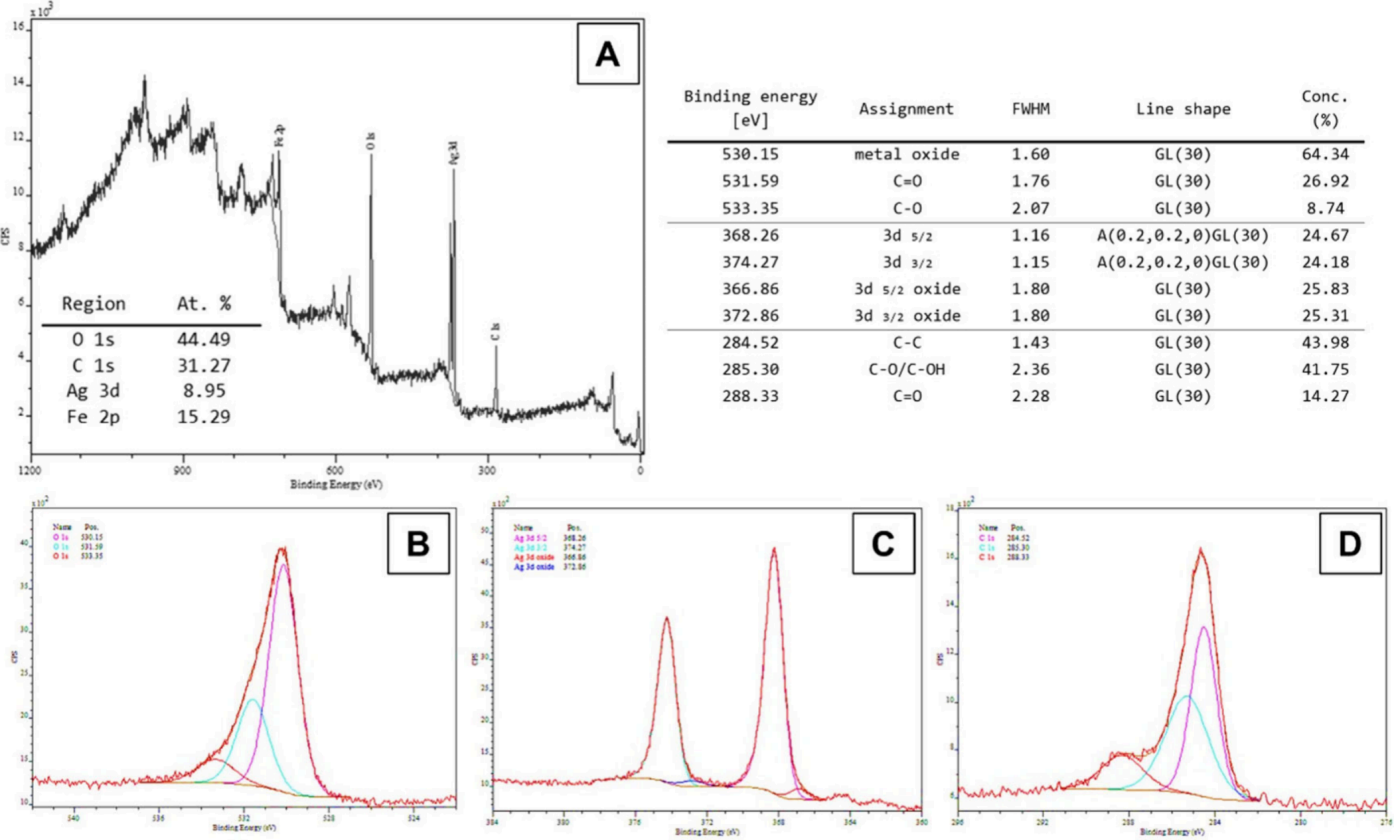
(A) X-ray photoelectron spectroscopy (XPS) wide-scan spectrum of Ag-NP layer obtained at −5 V. (B–D) Deconvolution XPS peaks of regions O 1s, Ag 3d, and C 1s. The table presents surface chemical compositions of AgNPs. Data processing was performed using the CasaXPS program; a Shirley baseline was used to cut off the background, and the signals were decomposed into mixed Lorentz and Gaussian lines. In the case of metallic states, an asymmetric function was used for the simulation. Multiplet structures were simulated based on the models included in ref (23).

**Figure 3:**
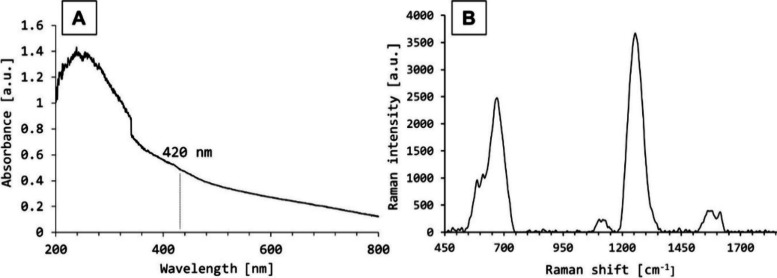
(A) UV–vis DRS spectrum and (B) Raman spectrum of the AgNP layer.

X-ray photoelectron spectroscopy (XPS) examinations of the layer’s surface indicated it is composed of metallic silver grains (Figure 2) with characteristic peaks such as C 1s, O 1s, and Ag 3d being observed. The Ag 3d_5/2_ and Ag 3d_3/2_ peaks appeared at binding energies of 368.26 and 374.27 eV, respectively, and the 6 eV splitting of the Ag 3d doublet confirms the formation of metallic silver.^22^ High-resolution analysis of the Ag 3d_5/2_ and Ag 3d_3/2_ peaks indicates the potential presence of Ag 3d oxide traces (Figure 2C), which may be attributed to factors such as residual undegraded precursor materials or incompletely decomposed trifluoroacetic acid species (Figure 2D). The ultraviolet–visible diffuse reflectance spectroscopy (UV–vis DRS) spectrum of the silver nanoparticles deposited on steel substrates is shown in Figure 3A. The absorption spectrum displays a subtle band at 420 nm, highlighting the need for careful interpretation. The formation of nanoparticles involves concurrent nucleation and growth of crystals, a critical aspect to consider when analyzing the spectrum. Raman spectroscopy data (referenced in Figure 3B) reveal the presence of signals at around 670 and 1255 cm^–1^. These findings imply that the laser radiation utilized in laser desorption/ionization (LDI) technology scatters off the silver nanoparticles, a phenomenon fundamental to the analysis of these signals.

### 3.2 Comparison of Background Signals in MALDI and AgNP-Based LDI

**Figure 4:**
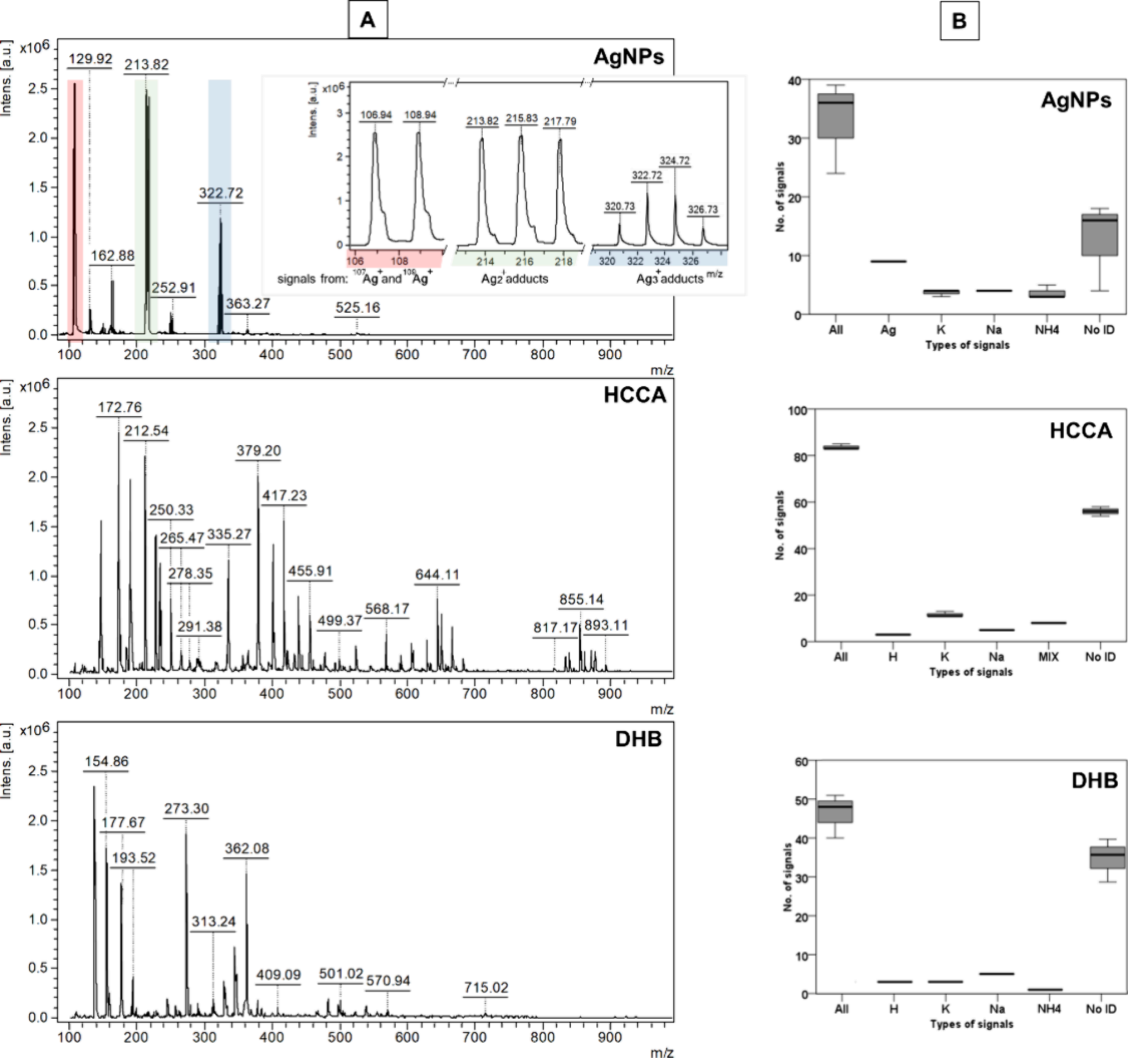
(A) Comparison of mass spectra generated by the surface covered with AgNPs and the matrices used (HCCA, DHB). (B) Box plot showing the average number (*n* = 3) of all signals recorded in individual mass spectra, the identified types of adducts with Na^+^, K^+^, NH_4_^+^, H^+^, or with at least two different ions (MIX), and not identified signals (No ID).

Mass spectra of the synthesized AgNP layer and the most commonly utilized matrices in the MALDI technique were recorded across an *m*/*z* range of 100–2000 in positive ion mode (refer to Figure 4). On average, the AgNPs produced 33 (±8) signals, a figure markedly lower than that observed for standard matrices: HCCA (α-cyano-4-hydroxycinnamic acid) generated 64 (±8) signals, and DHB (2,5-dihydroxybenzoic acid) produced 46 (±6) signals. Notably, for the majority of signals from AgNPs, the intensity was below 20% of the most intense signal (*m*/*z* = 215.83). A similar pattern was observed for DHB, whereas for HCCA, the intensity of most signals reached at least 20% of the highest signal intensity. The signals emanating from the nanoparticles were predominantly identified as clusters of silver isotopes (^107^Ag and ^109^Ag) and their adducts with Na, K, and NH_4_ ions (detailed in Figure 4B, with further information in Supplementary Table S1). In contrast, the organic matrices demonstrated a propensity for cluster formation (including 2M, 3M, 4M, M + [alkali ions], etc., where M represents the matrix) and fragmentation (M – H_2_O, M – OOH, etc.), resulting in fewer than half of all signals being identified (as listed in Supplementary Table S1).

### 3.3 Qualitative Analysis of Standards via NALDI

To assess its potential for detection, the AgNP layer, synthesized through the CVD technique, was tested with the LDI technique by recording mass spectra of standards. Low-molecular-weight compounds belonging to various classes of lipids, fatty acids, saccharides, amino acids, nucleosides, and carboxylic acids were used as standards.

**Figure 5:**
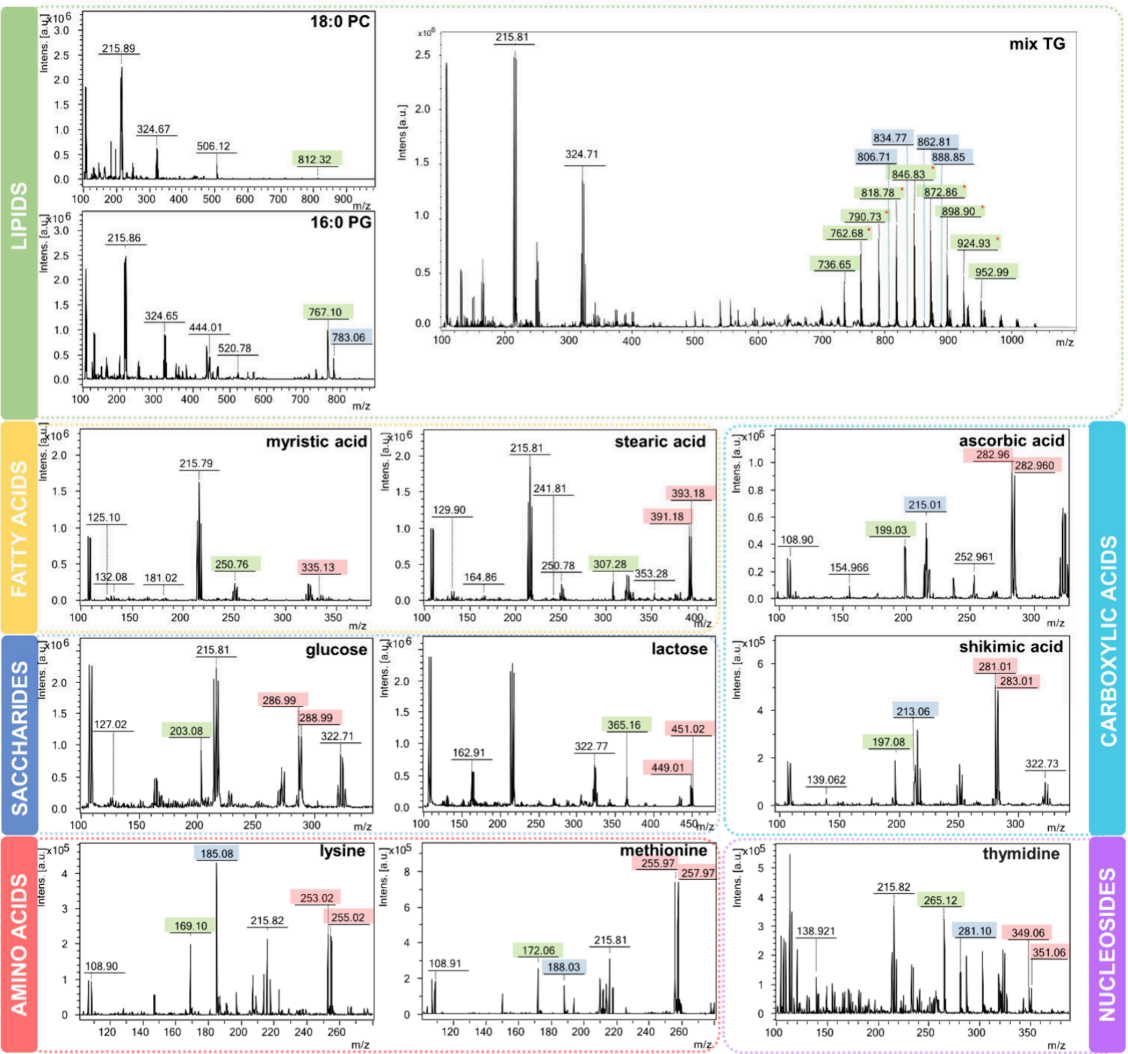
MS spectra recorded for the tested standards with the use of AgNPs synthesized by the CVD technique. The colors indicate the signals coming from the adducts of the analyzed compounds with sodium ions (green), potassium (blue), and silver isotopes (red); a red asterisk (*) reports that this analyte was also identified with a single ^13^C isotope.

Figure 5 displays the recorded spectra along with identified signals arising from adducts of the analyzed compounds with other ions. Notably, none of the analyzed compounds exhibited an adduct with a proton, which is a characteristic feature of the standard MALDI technique. Instead, all of the analyzed compounds appeared in the spectra as adducts with sodium ions, albeit with varying signal intensities. In comparison to internal silver signals originating from the plate surface, the most intense sodium adducts were recorded for amino acids, thymidine, and carboxylic acids, while the lowest were recorded for fatty acids and PC. In the case of TGs and sugars, the intensity of these signals was approximately half of the intensity of the silver signals. However, it is important to note that the initial concentration of triglycerides was significantly lower, ranging from 8 to even 40 times less than the concentrations of the other standards. Adducts with potassium ions were present in the spectra of compounds except for PC. However, in some cases, they were barely noticeable against the background noise. The intensity of the potassium signals was lower than that of the sodium signals, except for lysine. For TGs, signals of analytes with a single ^13^C isotope were also observed. Silver ion adducts (both isotopes ^107^Ag and ^109^Ag) were detected in all analyzed standards, excluding lipid standards. Their intensity varied: in the case of fatty acids, it was significantly higher for stearic acid; in the case of sugars, for the monosaccharide glucose; and in the case of amino acids, for the sulfur-containing methionine.

In the presented study, fragmentation spectra were recorded for lipid molecules (triglycerides, phospholipids, and fatty acids). Supplementary Table S2 depicts the identified molecular fragments of triglycerides, phospholipids, and fatty acids containing sodium, silver, and potassium adducts. The analysis encompasses various types of adducts, facilitating a comprehensive understanding of the ionization and fragmentation processes of these compounds in mass spectrometry techniques.

In the realm of triacylglycerol species, a diverse array exists, each characterized by the total length and saturation level of its acyl chains. Isobaric TGs, while sharing the same number of carbon atoms and double bonds, exhibit variations in the length, position, and configuration of their acyl chains. These chains occupy three distinct positions within the TG structure, denoted as the *sn*-1, *sn*-2, or *sn*-3 positions. Regiospecific analysis is primarily concerned with determining the orientation of the acyl chains at the *sn*-1(3) and *sn*-2 positions. Mass spectral fragmentation emerges as a valuable tool in elucidating the stereochemistry and regiospecific identification of TGs.^24^

**Figure 6:**
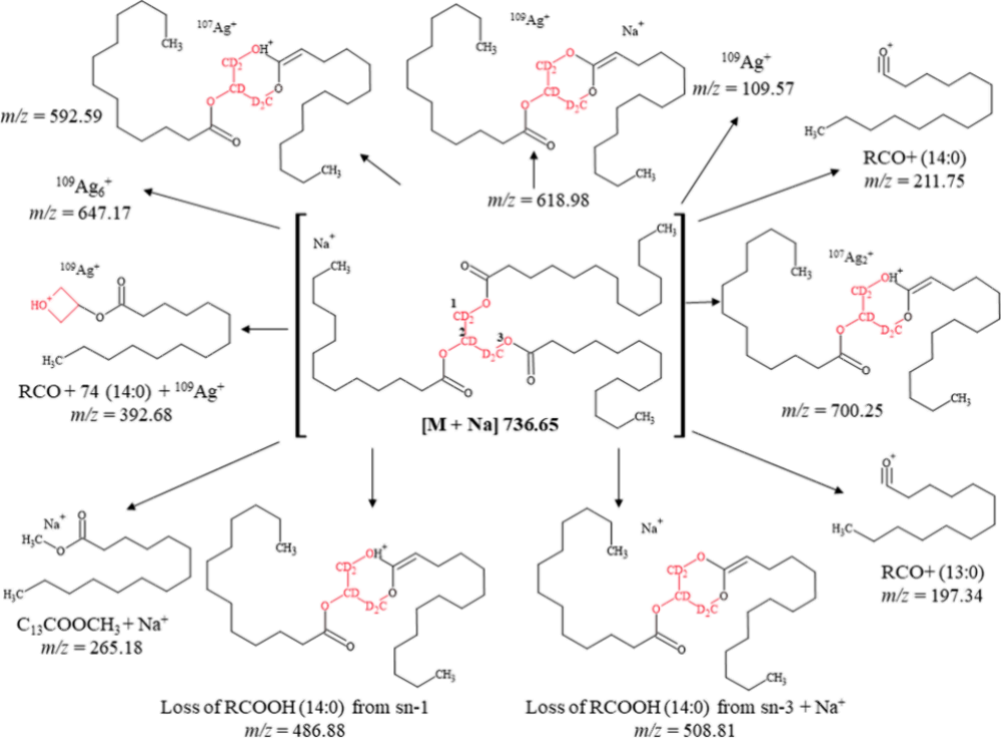
Fragmentation pathway of the sodium adduct of 14:0-13:0-14:0 TG-d5.

Figure 6 depicts the proposed fragmentation pathway of the sodium adduct of the triglyceride molecule [M + Na]^+^ at *m*/*z* = 736.65, corresponding to 14:0-13:0-14:0 TG-d5. Molecular ions underwent fragmentation, resulting in an peak at *m*/*z* = 486.88, corresponding to the loss of the 14:0 fatty acid as [M – R_1_COO]^+^, located at the *sn*-1 position. The loss of fatty acids at the *sn*-2 position is energetically less favorable compared to at the *sn*-1 and *sn*-3 positions, attributed to molecular steric properties.^24,25^ Additionally, a peak at *m*/*z* = 508.81 was observed, corresponding to the loss of the 14:0 fatty acid located at the *sn*-3 position and the attachment of a sodium ion [M – R_3_COO + Na]^+^. Fragments identified at *m*/*z* = 197.34 and 211.75 correspond to the acyl group of the tridecanoic acid ion [R_2_CO]^+^ and the acyl group of the myristic acid ion [R_1_CO]^+^, respectively. Furthermore, a peak at *m*/*z* = 265.18 was observed, corresponding to the molecular sodium adduct of the methyl ester of myristic acid [C_13_COOCH_3_ + Na]^+^. The ion detected at *m*/*z* = 392.68 can be described as the [R_1_CO + 74 + ^109^Ag]^+^ ion, corresponding to the acyl group of myristic acid and glycerol (mass 74 corresponds to the C_3_H_6_O_2_ molecule), along with the ^109^Ag adduct. Silver-containing molecular fragment adducts were also observed in the spectrum: *m*/*z* = 592.59 [M – R_1_COO + ^107^Ag]^+^, *m*/*z* = 618.98 [M – R_1_COOH + ^109^Ag + Na]^+^, and *m*/*z* = 700.25 [M – R_1_COOH + ^107^Ag_2_]^+^, resulting from bond cleavage upon laser irradiation of the triglyceride molecule. Additionally, silver ions at *m*/*z* = 109.57 and 647.17 were observed in the NALDI spectrum, corresponding to ^109^Ag^+^ and ^109^Ag_6_^+^, respectively. The presence of silver ions is induced by laser irradiation, enabling silver nanoparticles to replace commonly used protonating agents in MALDI-MS.^25^

**Figure 7:**
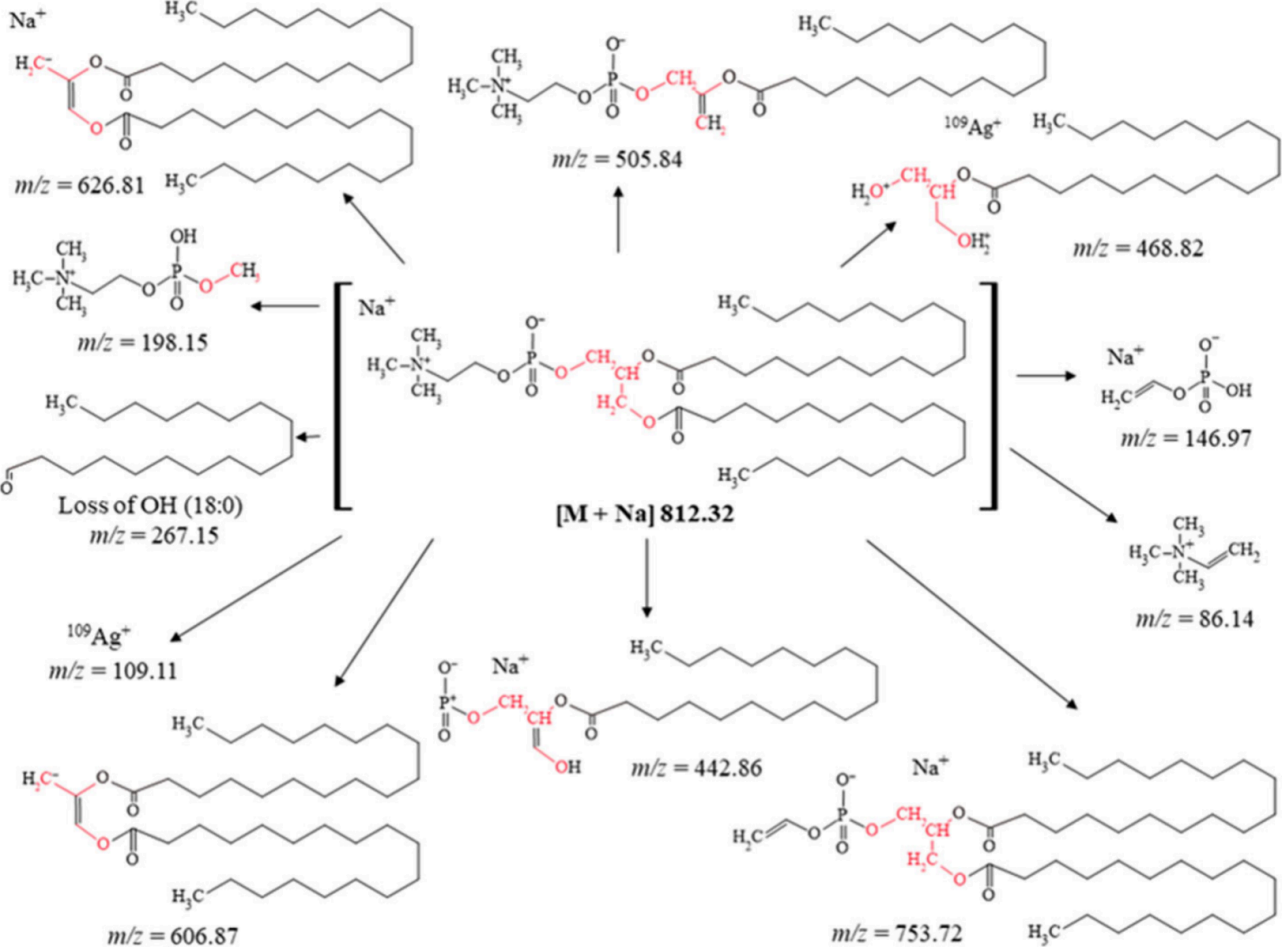
Fragmentation pathway of the sodium adduct with PC 18:0-18:0.

As depicted in Figure 7, the [M + Na]^+^ ion of PC 18:0-18:0 was detected at *m*/*z* = 812.32. Additionally, the spectrum exhibited the ion at *m*/*z* = 267.15, corresponding to the moiety of stearic acyl [M + RCO]^+^. In MALDI mode, the most characteristic ion of lipid compounds containing choline in their structure was observed at *m*/*z* = 184, corresponding to choline phosphate [C_5_H_15_NPO_4_]^+^. This ion serves as a “fingerprint” of molecules in the PC class.^26,27^ Through NALDI, phosphatidylcholine fragmentation was achieved. Although the ion at *m*/*z* = 184 was not observed in the spectrum, a prominent fragment at *m*/*z* = 198.15 appeared, which likely constitutes a characteristic fragment for lipid compounds containing choline. The ion at *m*/*z* = 198.15 corresponds to methyl phosphorylcholine [C_6_H_16_NPO_4_]^+^. The other ions at *m*/*z* = 146.97 and 86.14 correspond to sodium adduct with ethenyl hydrogen phosphate [C_2_H_4_PO_4_ + Na]^+^ and “dehydrocholine” [C_5_H_12_N]^+^. The small intense signal at *m*/*z* = 442.86 probably corresponds to the loss of “dehydrocholine” and moiety of stearic acyl and connection of the sodium adduct [C_21_H_40_PO_6_ + Na]^+^. Also observed in the fragmentation mass spectra was a fragment equal to *m*/*z* = 468.82, which probably corresponds to the silver adduct [C_21_H_44_O_4_ + ^109^Ag]^+^. The ion at *m*/*z* = 505.84 corresponds to [M + H – C_18_H_36_O_2_]^+^. Furthermore, the *m*/*z* ions 606.87 and 628.80 corresponding to [M – C_5_H_15_NPO_4_]^+^ and [M – C_5_H_15_NPO_4_ + Na]^+^ resulted from the neutral loss of methyl phosphorylcholine and the neutral loss of methyl phosphorylcholine with the connection of the sodium adduct, respectively. The peak at *m*/*z* = 753.72 corresponds to [M – C_3_H_10_NP + Na]^+^ (loss of trimethylamine).

### 3.4 Comparison of AgNP-Assisted LDI and MALDI in TG Analysis

The matrix-assisted laser desorption/ionization technique employs organic compounds as matrices to mediate energy transfer to the analyte, thus aiding in its ionization. However, this approach results in the mass spectrum being crowded with numerous matrix-derived signals, particularly in the lower measurement ranges. This complexity hampers the method’s effectiveness for analyzing low-molecular-weight compounds, such as lipids and sugars.

**Figure 8:**
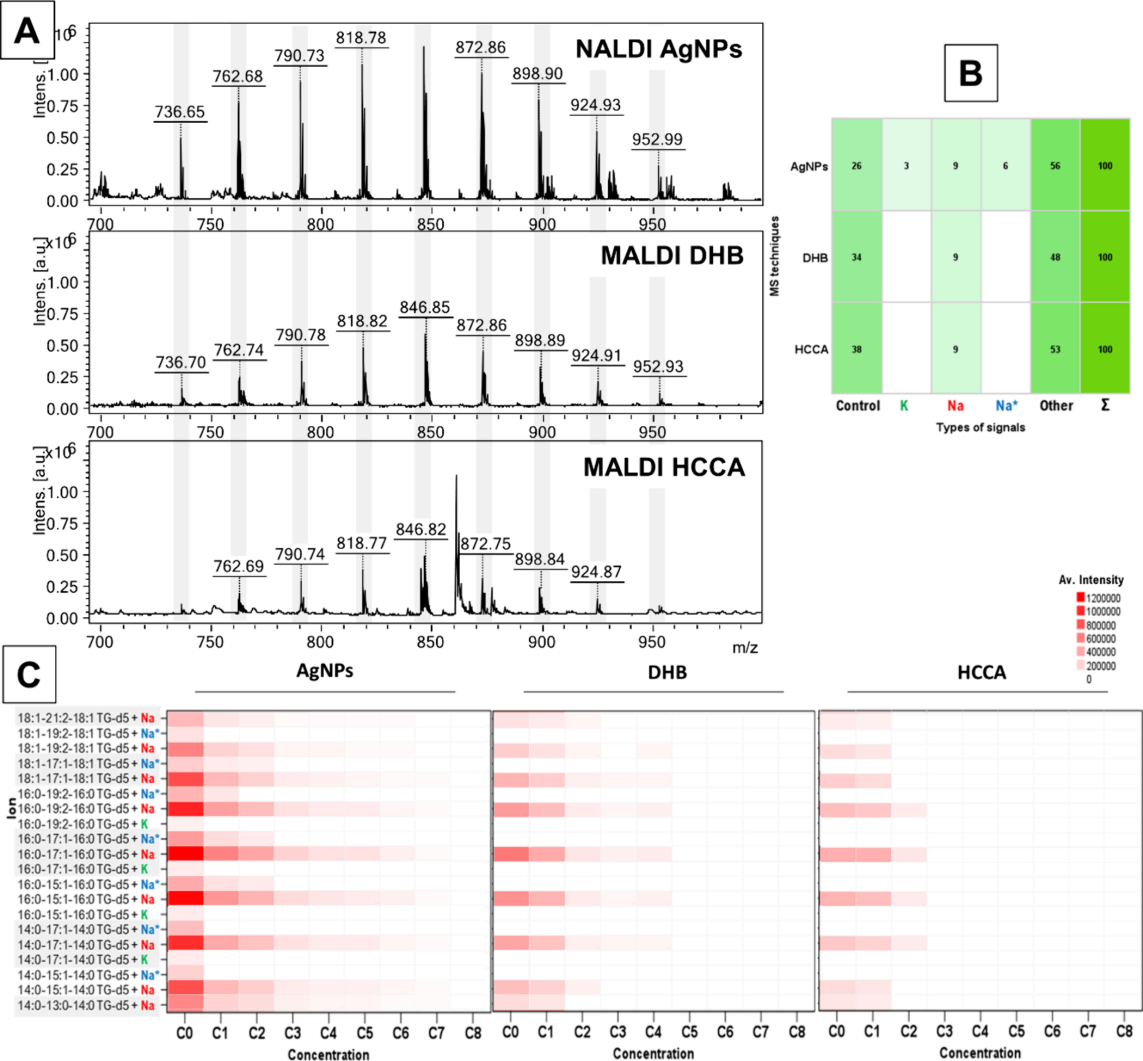
(A) Mass spectra in the *m*/*z* range of 700–1000 recorded for a mixture of triglyceride standards (initial concentration, *C*0) using AgNP- and matrix-assisted LDI techniques. (B) Average number of signals (*n* = 4) appearing in the recorded spectra of the TG mix standard, divided into those coming from nanoparticles or matrix (Control), adducts with ions, and unidentified ones (Other). (C) Average intensity of signals (*n* = 4) originating from triglyceride adducts with Na and K ions in spectra recorded using AgNPs and HCCA and DHB matrices; asterisks (*) mark compounds containing ^13^C.

To evaluate the comparative utility of the MALDI technique against the silver nanoparticle (AgNP) layer synthesized via the chemical vacuum deposition (CVD) technique, a mixture of triglycerides was analyzed, with various adducts observed in the NALDI spectra within the *m*/*z* range of 700–1000. Mass spectra for dilutions of triglyceride mix standards were recorded using both NALDI and MALDI techniques. Two matrices were employed: α-cyano-4-hydroxycinnamic acid (HCCA) and 2,5-dihydroxybenzoic acid (DHB). Figure 8A presents a comparison of the mass spectra for the triglyceride mixture at its initial concentration, obtained via AgNP-assisted and matrix-assisted LDI techniques.

The intensity of signals derived from triglyceride (TG) sodium adducts in the spectra obtained via silver nanoparticles demonstrated a substantial increase, approximately 50% greater than that obtained with the DHB matrix and about 70% higher compared to that obtained with HCCA. Furthermore, the analysis utilizing nanoparticle-assisted techniques also uncovered the formation of additional types of adducts, including those between the analytes and potassium, and complex adducts featuring a single ^13^C isotope bound to sodium, as illustrated in Figure 8B. Such adduct signals were notably absent in the spectra derived from conventional matrix-assisted methods. A significant proportion of the signals, particularly those within the *m*/*z* range of 300–1000, remained unidentified. These signals are hypothesized to originate either from adducts of triglyceride fragments with various ions, observed in both MALDI and NALDI spectra, or from fragments of the matrix itself in the case of MALDI spectra. Notably, the NALDI method, characterized by fewer identified signals within this range (on average about 8, all below *m*/*z* = 350), suggests a cleaner spectral profile with fewer interferences from nonanalyte-related ions. These signals were classified as control signals and generated directly by the AgNP-coated plate. The use of organic matrices significantly influenced the number of detectable control signals, with HCCA suppressing signals up to *m*/*z* = 1200, and DHB effectively reducing visibility up to approximately *m*/*z* = 540. In terms of S/N ratios, all unidentified signals in the nanoparticle-assisted techniques generally exhibited S/N ratios below 30–40, similar to those observed with the DHB matrix. In contrast, the HCCA matrix occasionally produced signals with markedly higher S/N ratios, in some instances exceeding values of 100.

Figure 8C illustrates a comparative analysis of signal intensities obtained from successive dilutions of the triglyceride mixture. The incorporation of nanoparticles substantially enhanced the detection sensitivity, facilitating the identification of lower triglyceride concentrations compared to those achieved using the traditional MALDI technique. Specifically, the use of the DHB matrix demonstrated greater sensitivity than the HCCA matrix. Calibration curves were constructed to represent the linear relationship between signal intensity and analyte concentration (nine concentrations; for *m*/*z* = 846 in the range from 40 ng/mL to 125 μg/mL) based on the signals from triglyceride sodium ion adducts. The regression coefficients for each TG and method are detailed in Table 2. For the DHB matrix, a linear correlation (defined by at least five data points) was established for five out of the nine tested TGs, with each correlation showing a regression coefficient consistently below 0.91. Conversely, for the HCCA matrix, it was impossible to plot linear relationships due to having fewer than five data points (see Supplementary Figure S1 in the Supporting Information). In contrast, the application of the AgNP-assisted method revealed a strong linear relationship, with *R*^2^ values of 0.986 or higher, indicating a robust correlation between the signal intensity and analyte concentration. These findings suggest that the tested AgNP layer offers increased sensitivity in measurements compared to traditional MALDI matrices, which influenced the ability to plot linear relationships in low concentration ranges (<125 μg/mL) of TGs.

**Table 2:** Regression Coefficients Calculated for the Plotted Relationship between Signal Intensity and Triglyceride Concentration^a^

		*R*^2^		
triglyceride	M + [adduct]	AgNPs	DHB	HCCA
18:1-21:2-18:1 TG-d5	929.85 + [Na]	0.986	-	-
18:1-19:2-18:1 TG-d5	901.81 + [Na]	0.990	-	-
18:1-17:1-18:1 TG-d5	875.80 + [Na]	0.996	0.905	-
16:0-19:2-16:0 TG-d5	849.78 + [Na]	0.997	0.899	-
16:0-17:1-16:0 TG-d5	823.77 + [Na]	0.993	0.889	-
16:0-15:1-16:0 TG-d5	795.74 + [Na]	0.997	0.882	-
14:0-17:1-14:0 TG-d5	767.71 + [Na]	0.996	0.866	-
14:0-15:1-14:0 TG-d5	739.67 + [Na]	0.992	-	-
14:0-13:0-14:0 TG-d5	713.66 + [Na]	0.994	-	-

a No *R*^2^ value corresponds to insufficient points on the curve to plot it.

### 3.5 Quantitative Analysis of Standards via NALDI

The analysis revealed that CVD-synthesized silver nanoparticles (AgNPs) exhibited variable ionization capabilities, which influenced the detection sensitivity of the analyzed standards. To facilitate quantitative analysis, we selected compounds that formed relatively intense molecular adducts; specifically, sodium adducts for triglycerides and potassium or silver adducts for other standards. The quantitative NALDI analysis enabled by these AgNPs was employed to obtain mass spectra from sample dilutions. Based on these spectra, we generated calibration curves to demonstrate the linear relationship between the signal intensity and analyte concentration. The calibration process incorporated eight data points for triglycerides and 5–7 data points for other standards, ensuring uniform coverage across the concentration range (refer to Table 3 for detailed data). Of the 15 tested analytes, 29 linear correlations were evaluated. Notably, 25 out of these 29 correlations yielded regression coefficients exceeding 0.99, underscoring a robust linear relationship between the concentration and detected intensity. The enhanced ionization efficiency afforded by the AgNPs was particularly evident in the analysis of triglycerides compared to other compounds. Triglycerides and methionine exhibited significantly lower limits of detection (LOD) and quantification (LOQ), with values ranging in the nanogram per milliliter scale, notably lower than those observed for other standards, which were in the microgram per milliliter range. Specifically, the lowest recorded values for LOD and LOQ were 10 ± 3 and 17 ± 5 ng/mL, respectively, for the triglyceride 14:0-13:0-14:0 TG-d5 (sodium adducts). In contrast, the highest LOD and LOQ recorded were 6.030 ± 1.614 and 10.050 ± 2.689 μg/mL, respectively, for shikimic acid with a potassium adduct.

**Table 3:** Details of the Correlation between the Signal Intensity and the Concentration of the Tested Standards Obtained Using the LDI Technique Supported by CVD-Synthesized Silver Nanoparticles

compound	analyte + [adduct]	linearity range (μg/mL)	*R*^2^	curve equation	LOD^a^ ± SD (μg/mL)	LOQ^b^ ± SD (μg/mL)
14:0-13:0-14:0 TG-d5	713.66 + [Na]^+^	0.04–25	0.9942	*y* = 19898*x* + 13693	0.010 ± 0.003	0.017 ± 0.005
14:0-15:1-14:0 TG-d5	739.67 + [Na]^+^	0.08–50	0.9924	*y* = 14712*x* + 22858	0.014 ± 0.006	0.023 ± 0.009
14:0-17:1-14:0 TG-d5	767.71 + [Na]^+^	0.12–75	0.9957	*y* = 11581*x* + 37044	0.014 ± 0.004	0.024 ± 0.006
16:0-15:1-16:0 TG-d5	795.74 + [Na]^+^	0.16–100	0.9966	*y* = 10395*x* + 48734	0.014 ± 0.005	0.023 ± 0.008
16:0-17:1-16:0 TG-d5	823.77 + [Na]^+^	0.20–125	0.9933	*y* = 8539.1*x* + 76230	0.012 ± 0.003	0.021 ± 0.005
16:0-19:2-16:0 TG-d5	849.78 + [Na]^+^	0.16–100	0.9968	*y* = 9337.8*x* + 36694	0.019 ± 0.007	0.031 ± 0.011
18:1-17:1-18:1 TG-d5	875.80 + [Na]^+^	0.12–75	0.9964	*y* = 10225*x* + 6499.6	0.026 ± 0.009	0.043 ± 0.015
18:1-19:2-18:1 TG-d5	901.81 + [Na]^+^	0.08–50	0.9902	*y* = 10789*x* – 1680.9	0.027 ± 0.001	0.044 ± 0.02
18:1-21:2-18:1 TG-d5	929.85 + [Na]^+^	0.2–25	0.9861	*y* = 11698*x* – 3080.4	0.064 ± 0.020	0.107 ± 0.33
glucose	180.16 + [Na]^+^	4–500	0.9902	*y* = 934.12*x* + 31426	1.622 ± 0.081	2.703 ± 0.135
glucose	180.16 + [^107^Ag]^+^	8–500	0.9908	*y* = 1627.5*x* + 30079	3.582 ± 0.454	5.970 ± 0.757
glucose	180.16 + [^109^Ag]^+^	8–500	0.9847	*y* = 1543*x* + 37393	2.927 ± 1.060	4.878 ± 1.767
lactose	342.20 + [^107^Ag]^+^	8–500	0.9919	*y* = 661.71*x* + 25959	1.882 ± 0.700	3.137 ± 1.166
lactose	342.20 + [^109^Ag]^+^	8–500	0.9933	*y* = 558.38*x* + 22024	2.192 ± 0.466	3.653 ± 0.777
methionine	149.21 + [Na]^+^	4–500	0.9906	*y* = 544.57*x* + 11042	0.348 ± 0.059	0.580 ± 0.099
methionine	149.21 + [^107^Ag]^+^	4–500	0.9961	*y* = 970.29*x* + 3747.4	0.419 ± 0.138	0.698 ± 0.231
methionine	149.21 + [^109^Ag]^+^	4–500	0.9962	*y* = 947.22*x* + 4122.7	0.416 ± 0.135	0.693 ± 0.226
lysine	146.19 + [Na]^+^	20–500	0.9941	*y* = 353.66*x* + 31567	5.063 ± 1.255	8.439 ± 2.092
lysine	146.19 + [K]^+^	20–500	0.9909	*y* = 745.66*x* + 55103	2.827 ± 0.762	4.711 ± 1.269
lysine	146.19 + [^107^Ag]^+^	8–500	0.9929	*y* = 340.67*x* + 46851	3.333 ± 0.403	5.556 ± 0.672
lysine	146.19 + [^109^Ag]^+^	20–500	0.9975	*y* = 338.86*x* + 38867	4.296 ± 1.012	7.160 ± 1.686
thymidine	242.23 + [Na]^+^	4–500	0.9882	*y* = 625.25*x* + 31612	1.304 ± 0.143	2.174 ± 0.238
thymidine	242.23 + [K]^+^	8–500	0.9825	*y* = 736.63*x* + 27559	3.200 ± 0.508	5.333 ± 0.846
thymidine	242.23 + [^107^Ag]^+^	8–500	0.9916	*y* = 255.3*x* + 14818	2.286 ± 0.197	3.810 ± 0.328
thymidine	242.23 + [^109^Ag]^+^	8–500	0.9900	*y* = 240.08*x* + 13466	2.637 ± 0.355	4.396 ± 0.591
shikimic acid	174.15 + [Na]^+^	20–500	0.9926	*y* = 687.01*x* + 4765.8	5.099 ± 1.298	8.499 ± 2.163
shikimic acid	174.15 + [K]^+^	20–500	0.9939	*y* = 394.45*x* + 10399	6.030 ± 1.614	10.050 ± 2.689
shikimic acid	174.15 + [^107^Ag]^+^	20–500	0.9976	*y* = 1273.6*x* + 25107	3.380 ± 0.815	5.634 ± 1.358
shikimic acid	174.15 + [^109^Ag]^+^	20–500	0.9982	*y* = 1193.1*x* + 19924	3.053 ± 0.734	5.089 ± 1.224

a LOD: limit of detection calculated based on an S/N ratio of 3.

b LOQ: limit of quantification calculated based on an S/N ratio of 5. SD: standard deviation; *R*^2^: regression coefficient.

## 4 Discussion

For silver nanoparticles, surface plasmon resonance (SPR) manifests across a broad spectral range, extending from ultraviolet to microwaves. This wide-ranging SPR capability renders AgNPs exceptionally versatile, making them a popular choice in mass spectrometry for detecting various low-molecular-weight compounds. Their sensitivity can reach femtomolar levels for certain substances. Utilizing nanoparticles in laser desorption/ionization offers a novel strategy to overcome the limitations faced in traditional MALDI techniques, which rely on organic compounds as matrices. In this study, we employed chemical vapor deposition to fabricate a uniform layer of AgNPs on a steel plate surface. We then evaluated the effectiveness of this nanoparticle layer in LDI mass spectrometry using lipid, saccharide, amino acid, and carboxylic acid standards and compared the performance to that of conventional MALDI techniques. Organic matrices used in MALDI exhibit significant absorption coefficients at the MS laser wavelength (Nd:YAG, 355 nm), promoting their evaporation and facilitating the transport of analyte molecules into the gas phase. In this phase, ions (such as H^+^ and Na^+^) exchange between the matrix and the analyte, enabling the analysis of the latter. However, these matrices exhibit pronounced selectivity, especially toward polar compounds, which complicates the ionization and analysis of different types of compounds, including lipids, via MALDI. As a result, the selection of an appropriate matrix for each analyte type is largely empirical. Ideally, the chosen matrix should enable the acquisition of high-resolution spectra with excellent signal-to-noise (S/N) ratios while maintaining a moderate matrix background and minimizing analyte fragmentation.^28^ The challenges outlined stem significantly from the analysis of low-molecular-weight compounds, such as lipids, which due to their smaller mass absorb radiation at shorter wavelengths similar to the matrix, leading to increased fragmentation. Artifacts arising from in-source decay complicate or skew the interpretation of spectra, a notable issue in lipidomics, where fragments can exhibit various isomeric forms.^29^ In the MALDI technique, lipid ionization predominantly occurs through the formation of adducts with small cations, particularly sodium, which is ubiquitously present in the samples. This interaction lowers the threshold energy required for molecule ionization and predisposes many lipid classes to fragmentation, especially enhancing the formation of fragment ions related to polar head groups.^29,30^ The matrix is typically present in a concentration approximately a thousand times greater than that of the analyte. Consequently, fragments of the analyte may interact with matrix fragments within the ion source, further obscuring the signal interpretation. Additionally, the sequence in which the analyte and matrix are applied to the target plate, along with the homogeneity of the resultant cocrystals, significantly influences both the accuracy and repeatability of quantitative analyses.^28^ In this study, the two matrices most frequently utilized in positive ion mode, 2,5-dihydroxybenzoic acid (DHB) and α-cyano-4-hydroxycinnamic acid (HCCA), were employed to explore these phenomena.

Silver nanoparticles (AgNPs) are renowned for their adjustable properties, which can be finely tuned through various synthesis techniques to enhance their suitability for laser desorption/ionization (LDI) applications in mass spectrometry (MS). The synthesis methods predominantly influence their morphology and size, directly impacting their interaction with analytes during LDI-MS analysis. The widely adopted methods for AgNP synthesis include the chemical reduction of silver nitrate and electrochemical deposition on cathode surfaces. These approaches are favored due to their simplicity and cost-effectiveness; however, they frequently result in uncontrolled aggregation and the formation of heterogeneous nanoparticle structures, leading to uneven analyte coverage and poor measurement repeatability.^31−33^ While the introduction of stabilizers during synthesis can mitigate these effects, it often complicates the spectral analysis by introducing extraneous peaks and interfering with ionization processes. In contrast, more precise fabrication methods such as electron beam lithography and focused ion beam offer enhanced control over the produced structures but are less suited for routine applications due to their high operational costs and extended processing times.^34,35^ This study utilized CVD to synthesize AgNPs, balancing meticulous control over nanoparticle characteristics with operational practicality and thus producing substrates that are immediately ready for analyte application and subsequent analysis. The superior performance of CVD-synthesized AgNPs is demonstrated by comparing it with those of other nanoparticle deposition techniques. Yagnik et al. employed sputter coating to achieve a uniform layer of nanoparticles, which significantly minimized the occurrence of “sweet spots” and improved the detection sensitivity across a range of analytes including lipids.^36^ This is in agreement with our observations, where the uniformity and intensity of the signals were significantly enhanced compared to those obtained with traditional matrix-assisted laser desorption/ionization (MALDI) matrices. Prysiazhnyi et al. also reported effective utilization of sputter-coated AgNPs for analyzing drugs, sugars, and lipids, highlighting the versatility of AgNPs when applied via physical vapor deposition (PVD) methods.^37^ Further extending the comparative analysis, the recent study described by Dufresne et al. illustrates the application of a sputtered silver coating for high-resolution imaging mass spectrometry (IMS) of olefins in tissue sections.^38^ The optimization of the silver coating thickness was critical in minimizing ion suppression effects, particularly in tissues with high lipid content, such as the brain. This finding complements our results that underscore the importance of nanoparticle uniformity in enhancing the quality and reproducibility of LDI-MS outcomes. The utilization of gold (AuNPs) and silver nanoparticles (AgNPs) enhances mass spectrometry (MS) analyses by leveraging their unique surface plasmon resonance properties. Silver nanoparticles are favored for their cost-effectiveness and ease of deposition through CVD, making them suitable for routine. The study by Dufresne et al. demonstrated that combining sodium salts with a sputtered gold layer (Au-CBS) on tissue sections increased signal intensity 30-fold compared to conventional MALDI methods, highlighting the potential for synergistic effects when different metallic elements and ionic species are used together.^39^ However, the economic implications and physicochemical properties of precursors, including volatility, require careful consideration. Optimizing deposition parameters is essential to balance cost-efficiency with analytical performance. By addressing these factors, the CVD method can be adapted more widely without sacrificing the quality necessary for precise analytical applications.

In this study, we utilized the CVD method to deposit a uniform epitaxial layer of silver nanoparticles on a steel plate surface. The resulting AgNP layer was confirmed to consist of a single layer of uniformly dispersed structures, with each nanoparticle measuring approximately 33.5 ± 1.5 nm in diameter. X-ray photoelectron spectroscopy (XPS) analysis conducted under normal conditions 30 days postsynthesis verified the sustained metallic state of the nanoparticles.^35^ The surface composition revealed a silver content of 8.95%, indicating a remarkably thin layer. This thinness posed challenges for characterization, particularly via UV–vis diffuse reflectance spectroscopy (DRS), and hindered the determination of the band gap energy. Additionally, the substantial presence of oxygen (44%) and carbon (31%) on the surface likely reflects the adsorption of atmospheric O_2_ and CO_2_ by the nanoparticles, a factor that may contribute to surface oxidation over time (after 30 days, about 1% of silver oxide).^40^

Mass spectra obtained in the positive mode from the synthesized layer of AgNPs exhibited a reduced number of signals, potentially minimizing signal suppression issues commonly encountered during the analysis of low-molecular-weight compounds, in contrast with materials traditionally employed for this purpose. DHB and HCCA tend to form a large number of clusters and adducts with various ions, the general formula of which can be written as M_DHB_ = *n*M + *p*[M – H_2_O] – *x*H + *y*[alkali metal ion]; M_HCCA_ = *n*M – *x*H + *y*[alkali metal ion] (*n* = 0, 1, 2, 3, ...; *p, x*, *y* ≥ 0).^41^ In our investigation, we noted a significant presence of silver cluster ions (Ag^+^, Ag^2+^, and Ag^3+^) within the nanoparticle spectra. Additionally, adducts of sodium and, to a lesser extent, potassium were identified. This observation aligns with the findings of Guan et al.,^42^ who reported similar adduct formation patterns and ion preferences in their study using PVP-capped AgNPs for lipid analysis via MALDI-MSI. Our data indicate a marked affinity of fatty acids’ carboxyl groups for sodium ions, corroborating with predictions from theoretical frameworks like the modified Poisson–Boltzmann model, which incorporates a finite ion size of 8 Å to accommodate the hydrated Na^+^ cation diameter of 7.2–8.0 Å.^43^ Density functional theory (DFT) and molecular dynamics (MD) simulations further elucidate the stability and interaction energies between fatty acids and silver ions, showcasing the dynamic and, at times, transient nature of these interactions. Specifically, secondary oxidation of AgNPs during laser desorption/ionization, as explored in the DFT study by Gallegos et al.,^44^ determines the stability of compounds, whereas MD simulations reveal AgNPs’ clearance properties stemming from weak interactions with fatty acids. Moreover, the interaction energy between stearic acids and a single silver ion is quantified at −2.79 kcal/mol, which is notably lower (by an order of magnitude) than that with sodium ions, highlighting the specificity of these interactions.^45^ In the context of the silver cluster–stearic acid complex, molecular orbital (MO) analysis demonstrated a bonding interaction, which dissociated after 5 ps. This interaction was characterized by an orbital overlap between the stearic acid and the silver cluster when they were approximately 2.33 Å apart. The complex dissociated as the distance increased to 10.09 Å, underscoring the critical role of spatial proximity in the stability of these molecular interactions. Our findings demonstrate a preference for sodium adducts over silver in AgNP-assisted laser desorption/ionization of fatty acids, indicative of selective interactions that significantly influence analytical outcomes. AgNPs mitigate matrix interference challenges, enhancing the detection of low-molecular-weight compounds. Analytical efficacy was assessed using lipid standards (phosphatidylcholine, phosphatidylglycerol, triglycerides, myristic acid, stearic acid) and saccharides (glucose, lactose), primarily in the positive ion mode, which typically offers enhanced sensitivity.^46^ Metal adducts, predominantly with sodium and, to a lesser extent, potassium and silver, were consistently observed, with phosphatidylglycerol and triglycerides showing optimal ionization.

AgNPs are particularly effective in detecting neutral lipids, forming weak cationic complexes with alkenes and showing high selectivity for olefin-containing lipids like cholesterol and fatty acids.^47^ This selectivity is crucial for distinguishing triglycerides in complex lipid mixtures, often obscured by more abundant phosphatidylcholine species in organic matrix-assisted analyses.^48^ Additionally, triglycerides predominantly formed sodium adducts, underscoring the influence of the electrochemical potential on adduct formation. The stability of proton adducts was generally lower, leading to potential fragmentation before reaching the detector, a limitation not observed with sodium adducts.^49^ The influence of nanoparticle size and laser fluence on the release of silver adducts was noted,^50^ with larger nanoparticles or higher fluence enhancing the release of Ag^+^ complexes. The degree of silver ionization also depended on the surface density of nanoparticles,^51^ and the binding preference of silver to olefins was influenced by the structural attributes of the lipid molecules.^52^ These observations suggest that the electrochemical properties of the adducting metal ions play a critical role in the ionization process of different biomolecules.

The mechanism of ionization in LDI techniques using silver nanoparticles is a complex and not fully understood process, especially since it proceeds differently in positive and negative ionization modes. To explain the desorption process, the thermally driven mechanism commonly used in MALDI has been broadly applied in metallic nanoparticle-assisted LDI techniques. The laser energy disrupts the binding interaction between the sample and the surface of the silver plate, transitioning it into the gas phase.^11^ This possibility is related to the surface plasmon resonance occurring in nanoparticles, which is responsible for heating and a strong local electromagnetic field; both effects can facilitate the ejection of atoms or even small clusters. One of the latest theories describing the involvement of SPR postulates that in the positive mode, sodium ions act as a secondary positive charge carrier.^53^ Sodium ions are excited to a high-energy state and react with adjacent analytes to form adducts. Positively charged adducts are immediately transferred to the external field by electrostatic repulsion, and as a result, they reach the mass analyzer.

Generally, AgNPs act as photocatalysts, helping to desorb and ionize analytes. The intricate process involves resonance-excited nanoparticles enhancing their cross section by up to 10 times, leading to the localization of a substantial amount of light energy near the surface of plasmonic nanostructures. This energy dissipates through processes such as photon emission (light scattering) and the induction of electronic transitions in plasmonic metals (via radiation absorption). Following light excitation, electrons in metallic nanostructures undergo transitions (interband and intraband), creating energetic charge carriers known as hot carriers. These hot electrons, possessing ample energy, can migrate to all available unoccupied states, including molecules adsorbed on the nanoparticle surface, through an indirect charge transfer pathway. In the excited state, the chemical bond of the molecule may elongate or even dissociate, facilitating subsequent chemical transformations. However, if the energy deposited on the molecule is insufficient to overcome the reaction energy barrier, the excited adsorbate reverts back to the ground state.^6^ This theory poses an interesting hypothesis that the amount of energy absorbed by nanoparticles affects the possibility of ionization or potential degradation of the molecules. This explains why fragmentation was observed for some of the tested standards (PC), while for others, only ionization was observed (saccharides and amino acids). Triglyceride fragmentation in the present study occurred to a small extent, and the loss of one of the chains was mainly involved. Conversely, in the case of PC 18:0-18:0, fragmentation was more noticeable and followed the loss of an aliphatic chain or a choline-containing fragment.

Different classes of lipids have different detection limits in LDI methods, which are determined by the structure and charge of the functional groups included in them; some will be easily protonated (like PC) and will be sensitively detected as a positive ion, while others may be deprotonated and exist in the form of anions (like PA or fatty acids).^28^ This partially coincides with the results obtained in this study. Moreover, lipid analysis using the MALDI technique presents challenges, not only because of the matrix-related signals in the spectra but also due to the selectivity of the matrix for the detection of individual lipids. In positive ion detection, the DHB matrix is especially recommended for lipid analyses,^46,54^ explaining the superior results obtained for TGs with it compared to HCCA. The situation is similar in the case of the analysis of saccharides, whose ionization and detection capabilities also depend on their structure and the matrix used.^55^

The application of nanoparticles allowed us to obtain linear correlations (*R*^2^ > 0.99 for tested saccharides and TG standards) of signal intensity and concentration, which was not possible using the MALDI technique. The reasons for obtaining a reproducible signal can be found in the distribution of the factors responsible for ionization. The AgNP layer, synthesized through chemical vapor deposition, exhibited a homogeneous distribution of nanoparticles. Conversely, in the case of the MALDI technique, a nonhomogeneous mixture of analysis and matrix is observed during the drying, which results in a highly irreproducible signal. In the case of DHB, the poor spot-to-spot reproducibility is caused by the formation of nonhomogeneous, needle-shaped large crystals by this matrix. The literature provides evidence that the utilization of AgNPs enables quantitative analysis of low-weight molecules using the LDI technique, for instance, in the case of carboxylic acids,^14^ amino acids,^21^ or some medicines like trimethoprim.^51^ Our study also confirmed these findings for amino acids, nucleosides, and carboxylic acids, where we observed the presence of sodium, potassium, and silver adducts in the spectra and the ability to plot calibration curves with *R*^2^ > 0.99 for concentrations below 500 mg.

## 5 Conclusion

The detection of low-molecular-weight compounds remains a significant challenge in MALDI mass spectrometry. An approach that is becoming increasingly popular is the replacement of classic matrices with metal nanoparticles, which, thanks to their different optical properties, interact with laser radiation and enable sample ionization. This approach not only is beneficial due to significantly reduced matrix background signals but also provides greater selectivity, sensitivity, and effectiveness of analytical techniques. The proposed technique for silver nanoparticle synthesis via CVD showcased the capability to detect various low-molecular-weight compounds such as lipids, saccharides, amino acid, and carboxylic acids. The obtained nanostructures were characterized by high selectivity toward triglycerides, the analysis of which using MALDI is extremely difficult due to the ongoing fragmentation, which was insignificant in our studies. The obtained AgNPs, thanks to their uniform deposition on the surface of the plate, made it possible to plot linearity ranges, which is not possible using the MALDI technique. Supporting the ionization of an analyte by nanoparticles is an extremely difficult process to characterize; however, we believe that the plasmonic and photocatalytic properties of metallic nanoparticles play a significant role in it. These findings underscore the need for further exploration into controlling the size and deposition thickness of AgNP layers via techniques such as CVD and ALD. This ongoing research holds the promise of providing a deeper understanding of ionization mechanisms and has the potential to broaden the applications of NALDI techniques in the analysis of low-molecular-weight compounds in the field of biomedicine and industrial applications.

## Supporting Information Available

Masses of identified signals on mass spectra registered for CVD-synthesized AgNPs and standard MALDI matrices, HCCA and DHB; identified molecular fragments of triglycerides, phospholipids, and fatty acids containing sodium and silver adducts; and mass spectra recorded for various concentrations of triglyceride 16:0-15:1-16:0 TG-d5 (PDF)
