# Supplementary material for: Advances in LDI-MS Analysis: The Role of Chemical Vapor Deposition-Synthesized Silver Nanoparticles in Enhancing Detection of Low-Molecular-Weight Biomolecules
Source: J Am Soc Mass Spectrom. 2024 Aug 14;35(9):2041–55. doi: 10.1021/jasms.4c00071 (PMC11378275; doi:10.1021/jasms.4c00071)
Supplement: Supplementary file 1 — js4c00071_si_001.pdf [file js4c00071_si_001.pdf]

Supporting Information

Advances in LDI-MS Analysis: The Role of Chemical Vapor Deposition-Synthesized Silver Nanoparticles in Enhancing Detection of Low-Molecular-Weight Biomolecules

Ewelina Sibińska[a*], Justyna Walczak-Skierska[a], Adrian Arendowski[a], Agnieszka Ludwiczak[a,b], Aleksandra Radtke[c], Piotr Piszczek[c], Dorota Gabryś[d], Kinga Robotnik[a,c], Paweł Pomastowski[a,c]

[a] *Centre for Modern Interdisciplinary Technologies, Nicolaus Copernicus University in Toruń, Wileńska 4 Str., 87-100 Toruń, Poland*

[b] *Faculty of Biological and Veterinary Sciences, Nicolaus Copernicus University in Toruń, Lwowska 1 Str., 87-100 Toruń, Poland*

[c] *Department of Inorganic and Coordination Chemistry, Faculty of Chemistry, Nicolaus Copernicus University in Toruń, Gagarina 7 Str., 87-100 Toruń, Poland*

[d] *Radiotherapy Department, Maria Sklodowska-Curie National Research Institute of Oncology, Wybrzeże Armii Krajowej 15 Str., 44-102 Gliwice, Poland*

**corresponding author: e_maslak@doktorant.umk.pl*

Supplementary Table S1. Observed and theoretical masses of identified signals on mass spectra registered for AgNPs synthetized via CVD technique and standard MALDI matrices: HCCA, DHB.

AgNPs

Cluster composition		Molecular weight	
		observed	teoretical
**1.**	$^{107}Ag^{+}$	106.94	106.91
**2.**	$^{109}Ag^{+}$	108.94	108.90
**3.**	$^{107}Ag + NH_4^{+}$	124.99	124.94
**4.**	$^{109}Ag + NH_4^{+}$	126.98	126.94
**5.**	$^{107}Ag + Na^{+}$	129.92	129.89
**6.**	$^{109}Ag + Na^{+}$	131.92	131.89
**7.**	$^{107}Ag + K^{+}$	145.87	146.00
**8.**	$^{109}Ag + K^{+}$	147.87	148.00
**9.**	$^{107}Ag + Na^{+} + H_3O$	148.91	148.92
**10.**	$^{109}Ag + Na^{+} + H_3O$	150.90	150.92
**11**	$^{107}Ag + K^{+} + O$	162.88	162.00
**12.**	$^{109}Ag + K^{+} + O$	164.88	164.00
**13.**	$^{107}Ag_2^{+}$	213.82	213.81
**14.**	$[^{107}Ag + {}^{109}Ag]^{+}$	215.83	215.81
**15.**	$^{109}Ag_2^{+}$	217.79	217.81
**16.**	$^{107}Ag_2^{+} + NH_3 + H_2O$	248.85	248.86
**17.**	$[^{107}Ag + {}^{109}Ag]^{+} + NH_3 + H_2O$	250.94	250.86
**18.**	$^{109}Ag_2^{+} + NH_3 + H_2O$	252.88	252.86
**19.**	$^{107}Ag_3^{+}$	320.73	320.72
**20.**	$[^{107}Ag_2 + {}^{109}Ag]^{+}$	322.72	322.71
**21.**	$[^{107}Ag + {}^{109}Ag_2]^{+}$	324.72	324.71
**22.**	$^{109}Ag_3^{+}$	326.73	326.71
**23.**	$^{107}Ag + {}^{109}Ag_2 + K^{+}$	363.77	343.71
**24.**	$^{109}Ag_3 + K^{+}$	365.78	363.81

HCCA

Cluster composition		Molecular weight	
		observed	teoretical
**1.**	$M+H^{+}$	190.63	190.18
**2.**	$M+NH_4^{+}$	207.53	207.21
**3.**	$M+Na^{+}$	212.52	212.16
**4.**	$M+Na^{+}+H^{+}$	213.45	213.17
**5.**	$M+K^{+}$	228.38	228.27
**6.**	$M+K^{+}+2H^{+}$	230.37	230.28
**7.**	$M+2Na^{+}-H^{+}$	234.39	234.14
**8.**	$M+2Na^{+}$	235.56	235.15
**9.**	$M+Na^{+}+K^{+}-H^{+}$	250.32	250.25
**10.**	$M+2K^{+}-H^{+}$	266.22	266.36
**11**	$2M+H^{+}$	379.17	379.34
**12.**	$2M+Na^{+}$	401.13	401.33
**13.**	$2M+K^{+}$	417.22	417.43
**14.**	$2M+Na^{+}+K^{+}-H^{+}$	439.13	439.42
**15.**	$2M+2K^{+}$	455.90	456.53
**16.**	$3M+H^{+}$	568.16	568.13
**17.**	$3M+K^{+}$	606.10	606.09
**18.**	$3M+Na^{+}+K^{+}-H^{+}$	628.10	628.07
**19.**	$3M+2K^{+}-H^{+}$	644.11	644.05
**20.**	$3M+Na^{+}+2K^{+}-2H^{+}$	666.09	666.03
**21.**	$3M+3K^{+}-2H^{+}$	682.07	682.00
**22.**	$4M+2K^{+}-H^{+}$	833.13	833.09
**23.**	$4M+Na^{+}+2K^{+}-2H^{+}$	855.13	855.07
**24.**	$4M+3K^{+}-2H^{+}$	871.12	871.04
**25.**	$4M+Na^{+}+3K^{+}-3H^{+}$	893.12	893.03
**26.**	$5M+Na^{+}+2K^{+}-2H^{+}$	1044.14	1044.11
**27.**	$5M+3K^{+}-2H^{+}$	1060.13	1060.09
**28.**	$5M+Na^{+}+3K^{+}-3H^{+}$	1082.14	1082.07
**29.**	$5M+4K^{+}-3H^{+}$	1098.12	1098.04
**30.**	$6M+3K^{+}-2H^{+}$	1249.14	1249.13
**31.**	$6M+Na^{+}+3K^{+}-3H^{+}$	1271.13	1271.09

**32.**	$5M+4K^+-3H^+$	1287.11	1287.08

DHB

	Cluster composition	Molecular weight	
		observed	teoretical
**1.**	$M+H^+$	154.84	155.15
**2.**	$MH+H^+$	155.81	156.14
**3.**	$M-H20+Na^+$	159.78	159.09
**4.**	$M+Na^+$	177.65	177.11
**5.**	$M+K^+$	193.51	193.22
**6.**	$2M+NH_4^+-2H2O$	290.19	290.07
**7.**	$2M-2H2O+H^+$	273.29	273.22
**8.**	$2M-H2O+Na^+$	313.22	313.22
**9.**	$2M-H2O+K^+$	329.12	329.32
**10.**	$2M+Na^+$	331.06	331.23
**11**	$2M+(2Na)^+$	345.09	354.22
**12.**	$2M+K^+$	347.05	347.34

Supplementary Table S2. Identified molecular fragments of triglycerides, phospholipids, and fatty acids containing sodium and silver adducts.

**Compound**	**X + Na**	**X + Ag**	**Fragmentation ions**
14:0-13:0-14:0 TG-d5	265.18		$C_{15}H_{30}O_2 + Na$
		392.70	$C_{17}H_{33}O_3 + {}^{109}Ag$
	508.81		$C_{30}H_{51}D_5O_4 + Na$
		592.60	$C_{30}H_{51}D_5O_4 + {}^{107}Ag$
	618.99	618.99	$C_{30}H_{51}D_5O_4 + H + {}^{109}Ag + Na$
		700.26	$C_{30}H_{51}D_5O_4 + {}^{107}Ag_2$
	736.65		$C_{44}H_{79}D_5O_6 + Na$
14:0-15:1-14:0 TG-d5	263.73		$C_{15}H_{28}O_2 + Na$
		391.76	$C_{17}H_{33}O_3 + {}^{109}Ag - 2H$
	534.83		$C_{32}H_{53}D_5O_4 + Na$
		618.48	$C_{32}H_{53}D_5O_4 + {}^{109}Ag - H$
	762.68		$C_{46}H_{81}D_5O_6 + Na$
14:0-17:1-14:0 TG-d5		398.12	$C_{17}H_{27}D_5O_3 + {}^{109}Ag + H$
	561.83		$C_{34}H_{57}D_5O_4 + Na - H$
		647.14	$C_{34}H_{57}D_5O_4 + {}^{107}Ag - H$
	790.73		$C_{48}H_{85}D_5O_6 + Na$
16:0-15:1-16:0 TG-d5	561.85		$C_{34}H_{57}D_5O_4 + Na - H$
		647.16	$C_{34}H_{57}D_5O_4 + {}^{107}Ag - H$
	818.78		$C_{50}H_{89}D_5O_6 + Na$
16:0-17:1-16:0 TG-d5	347.80		$C_{20}H_{37}O_3 + Na - H$
	591.04		$C_{36}H_{61}D_5O_4 + Na$
	846.83		$C_{52}H_{93}D_5O_6 + Na$
16:0-19:2-16:0 TG-d5	317.10		$C_{19}H_{34}O_2 + Na$
	374.92		$C_{20}H_{35}O_3 + Na$
	617.00		$C_{38}H_{63}D_5O_4 + Na$
	724.99	724.99	$C_{38}H_{63}D_5O_4 + {}^{109}Ag + Na$
	872.86		$C_{54}H_{95}D_5O_6 + Na$
18:1-17:1-18:1 TG-d5	347.81		$C_{20}H_{36}O_3 + Na$
	617.00		$C_{38}H_{63}D_5O_4 + Na$
		702.62	$C_{38}H_{63}D_5O_4 + {}^{109}Ag$
	898.90		$C_{56}H_{97}D_5O_6 + Na$
18:1-19:2-18:1 TG-d5	317.93		$C_{19}H_{34}O_2 + Na$
	375.85		$C_{20}H_{35}O_3 + Na + H$
	592.91	592.91	$C_{20}H_{61}O_3 + {}^{109}Ag_2 + Na - H$
	643.02		$C_{40}H_{65}D_5O_4 + Na$
		727.90	$C_{40}H_{65}D_5O_4 + {}^{109}Ag$
	924.93		$C_{58}H_{99}D_5O_6 + Na$
18:1-21:2-18:1 TG-d5	363.88		$C_{21}H_{39}O_3 + Na + H$
	671.07		$C_{42}H_{69}D_5O_4 + Na + H$
		754.79	$C_{42}H_{69}D_5O_4 + {}^{107}Ag$

	952.99		$C_{60}H_{103}D_5O_6 + Na$
PG 16:0-16:0	221.10		$C_5H_{11}PO_6 + Na$
	507.56		$C_{22}H_{45}PO_9 + Na$
	767.10		$C_{38}H_{75}PO_{10} + Na$
	783.06		$C_{38}H_{75}PO_{10} + K$
PC 18:0-18:0	146.98		$C_2H_4PO_4 + Na$
	442.86		$C_{21}H_{40}PO_6 + Na$
		468.83	$C_{21}H_{44}O_4 + {}^{109}Ag$
	628.81		$C_{39}H_{73}O_4 + Na$
	753.73		$C_{41}H_{78}PO_8 + Na$
	812.32		$C_{44}H_{88}NPO_8 + Na$
$C_{13}H_{27}COOH$	250.76		$C_{13}H_{27}COOH + Na$
		335.13	$C_{13}H_{27}COOH + {}^{107}Ag$
		337.13	$C_{13}H_{27}COOH + {}^{109}Ag$
$C_{17}H_{35}COOH$	307.28		$C_{17}H_{35}COOH + Na$
		391.18	$C_{18}H_{35}COOH + {}^{107}Ag$
		393.18	$C_{18}H_{35}COOH + {}^{109}Ag$

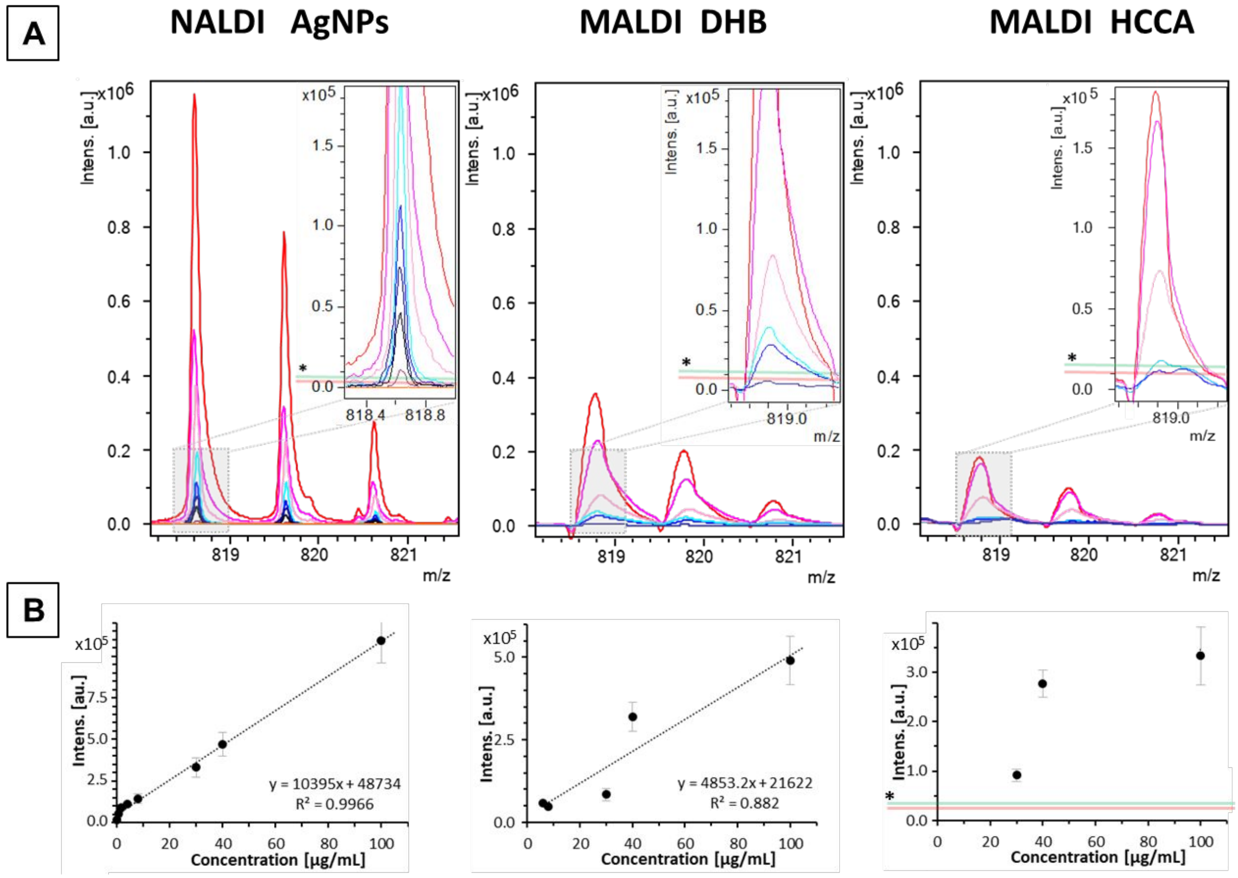

Supplementary Figure S1. A -Mass spectra recorded for various concentrations of triglyceride 16:0-15:1-16:0 TG-d5 (sodium adduct; *m/z* = 818.8) recorded using the LDI technique assisted by AgNPs and matrices (HCCA and DHB). The limit of detection (LOD) was determined based on S/N values. Signals for the same triglyceride concentrations (100, 40, 30, 8, 4, 1.6, 0.8, 0.16, 0.032 µg/mL) recorded using different LDI techniques are marked in colors. * green line corresponds to LOQ (S/N = 5), red line corresponds to LOD (S/N = 3). **B -** Calibration curves illustrating the dependence of the signal intensity of triglyceride 16:0-15:1-16:0 TG-d5 (*m/z* = 818.8) on their concentration, plotted based on spectra recorded using LDI techniques. In case of HCCA concentration below 8 ng/mL (4, 1.60, 0.80, 0.16, and 0.032 ng/mL) the intensity of signals was below LOD.
